# Expanding the scope of SGLT inhibitors in underrepresented cardiac populations: from pathophysiology to clinical evidence

**DOI:** 10.3389/fcvm.2026.1809193

**Published:** 2026-05-08

**Authors:** Banmeet Padda, Jane T. Kelleher, Valtteri Muroke, Vikas S. Sridhar, Maxime Tremblay-Gravel, Jean-Claude Tardif, Jacinthe Boulet

**Affiliations:** 1 Faculté de Médecine, Université de Montréal, Montreal, QC, Canada; 2 Harvard-MIT Program in Health Sciences and Technology, Harvard Medical School, Boston, MA, United States; 3 Department of Medicine, Division of Cardiology, Montreal Heart Institute, Université de Montréal, Montreal, QC, Canada; 4 Department of Medicine, Division of Nephrology, University of British Columbia, Vancouver, BC, Canada

**Keywords:** congenital heart disease, heart failure, heart transplant, hypertrophic cardiomyopathy, left ventricular assist device, sodium-glucose cotransporter 2 inhibitors, transthyretin amyloid cardiomyopathy

## Abstract

Sodium-glucose cotransporter inhibitors, originally developed for diabetes management, have demonstrated significant therapeutic benefit across the phenotypic spectrum of heart failure in landmark trials. However, multiple patient populations predisposed to ventricular dysfunction, including transthyretin amyloid cardiomyopathy, hypertrophic cardiomyopathy, congenital heart disease, heart transplant, and left ventricular assist devices, were initially excluded from these key trials due to their unique underlying cardiac physiology and limited safety data. Nonetheless, the pleiotropic effects of SGLTi, like modulation of inflammation, fibrosis, systemic venous congestion, endothelial dysfunction, and renal impairment, may also be advantageous in these patient populations. This narrative review discusses the potential role of SGLTi in these populations, primarily based on current observational evidence. We also examine emerging mechanistic insights from preclinical research and summarize ongoing clinical trials that may further clarify the therapeutic potential of SGLTi in these contexts. Finally, safety considerations, including monitoring volume status, the risk of euglycemic ketoacidosis, and genitourinary infections, are discussed. Ongoing prospective studies and interventional trials will be essential to better define the safety and efficacy of SGLTi in these underrepresented patient groups.

## 1 Introduction

Sodium-glucose cotransporters (SGLT) are a family of proteins primarily located in segments 1 and 2 of the proximal tubule of the kidney (SGLT2), or in enterocytes of the small intestine (SGLT1), and segment 3 of the proximal tubule of the kidney (SGLT1). SGLT in the kidney reabsorb over 90% of filtered glucose via a sodium-coupled mechanism (1). These cotransporters were initially targeted for plasma glucose reduction. In 2014, the first SGLT2 inhibitors (SGLT2i), dapagliflozin and empagliflozin, which target SGLT2, were approved for the treatment of type 2 diabetes mellitus (T2DM) (2, 3). Sotagliflozin, an SGLT1/2 dual inhibitor, was approved for the treatment of T2DM shortly after in 2018. Subsequently, meta-analyses of large-scale cardiovascular (CV) outcome trials demonstrated that SGLT2i were associated with significant reductions in major adverse CV events, heart failure (HF) hospitalizations, and progression of diabetic kidney disease in patients with T2DM and CV risk (4, 5). Similarly, SGLT1/2 dual inhibitors were associated with benefits in patients with T2DM and heart failure (6).

These findings motivated the HF landmark DAPA-HF and DELIVER trials using dapagliflozin, and EMPEROR-Reduced and EMPEROR-Preserved using empagliflozin, which demonstrated that SGLT2i reduce the risk of CV death and HF-related hospitalizations, regardless of the underlying left-ventricular function and diabetes status (7–10). As a result of this robust evidence supporting SGLT2i use in HF with reduced ejection fraction (HFrEF) independent of their glucose-lowering effects, the Canadian Journal of Cardiology (CJC), the American College of Cardiology (ACC), the American Heart Association (AHA), European Society of Cardiology (ESC), and the Heart Failure Society of America (HFSA) formally recommended SGLT2i as guideline-directed medical therapy (GDMT) for HFrEF in 2021 and 2022 (11, 12). Subsequently, SGLT2i were incorporated in the GDMT for HF with preserved ejection fraction (HFpEF), as endorsed by the ACC Expert Consensus Decision Pathway, the ESC focused update, as well as the CJC HFpEF guidelines (13–15). In addition, the SGLT1/2 dual inhibitor sotagliflozin reduced CV deaths and hospitalizations compared with placebo in the SOLOIST-WHF trial across the spectrum of HF. However, the current guidelines primarily recommend selective SGLT2 inhibitors, reflecting the larger body of randomized evidence supporting these agents (6).

However, the role of SGLT inhibitors (SGLTi) in several heart failure subgroups remains uncertain. Many of these populations, including patients with infiltrative cardiomyopathies, hypertrophic cardiomyopathy (HCM), prior heart transplantation (HTx), and complex congenital heart disease, were largely excluded from major randomized clinical trials (6–10). As a result, the available evidence in these groups is limited and derived predominantly from retrospective and observational studies.

The mechanisms underlying the clear clinical benefits of SGLTi in HF remain an area of active investigation. Likely avenues include insulin-independent glucosuria, the cardiorenal benefits of SGLTi may be attributable to volume reduction via natriuresis and osmotic diuresis, favorable modulation of inflammatory pathways, mitochondrial function, and potential anti-arrhythmic effects (16–18). While these mechanisms are better characterized in SGLT2 inhibitors, dual SGLT1/2 inhibitors show similar mechanistic and clinical effects (19, 20).

**Figure 1:**
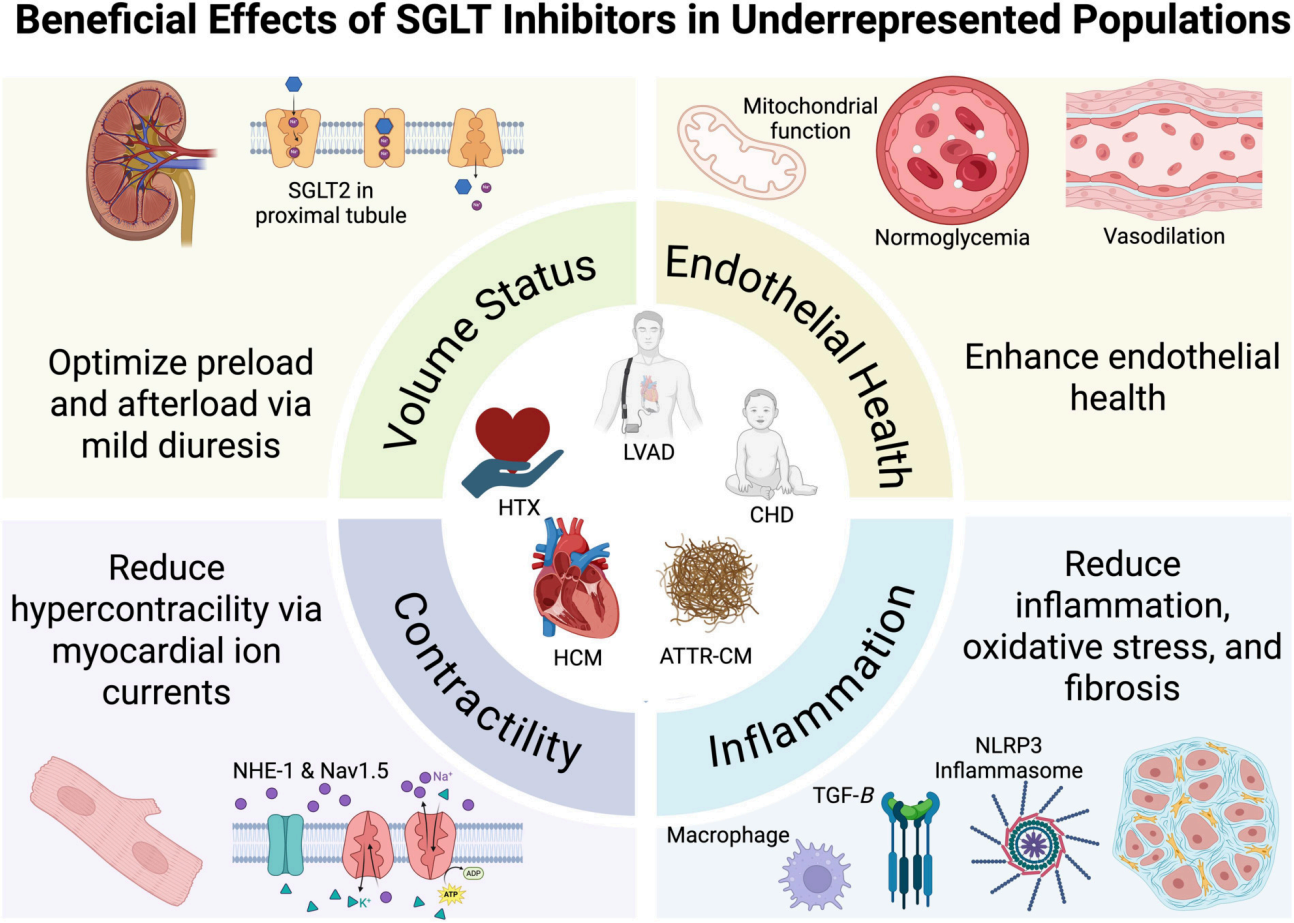
Beneficial effects of SGLT inhibitors in underrepresented populations. Created with BioRender.

These multifaceted mechanistic pathways support the potential of SGLTi, including both SGLT2i and SGLT1/2 dual inhibitors, to improve clinical outcomes across previously excluded cardiac populations in the previous HF landmark trials (8, 9). Herein, we review the potential role of SGLTi in the care of patients with transthyretin amyloid cardiomyopathy, HCM, congenital heart disease (CHD), HTx, and left ventricular assist device (LVAD) (Figure 1). Although other underrepresented heart failure populations exist, these groups were chosen for this review because they represent clinically important subgroups with distinct cardiac physiology that were excluded from major randomized trials.

## 2 Methods

This is a narrative review of the emerging use of SGLTi in patient populations not represented in major clinical trials, specifically amyloid cardiomyopathy, HCM, CHD, HTx, and LVAD. Literature searches were conducted in PubMed/MEDLINE and ClinicalTrials.gov up to November 2025. Search terms included “SGLT2 inhibitor,” “SGLT1/2 inhibitor,” “Empagliflozin,” “Dapagliflozin,” “Canagliflozin,” “amyloid cardiomyopathy,” “hypertrophic cardiomyopathy,” “hypertrophic obstructive cardiomyopathy,” “congenital heart disease,” “heart transplant,” “left ventricular assist device,” “LVAD,” and their combinations.

Additional candidate articles were identified by screening the reference lists of retrieved articles and conducting targeted searches in Google Scholar using the same search terms. Titles and abstracts were screened by the authors, and those considered potentially relevant were reviewed in full. Pertinent findings were extracted and discussed narratively using thematic grouping with the goal of highlighting SGLTi mechanism, the potential benefits and drawbacks of SGLTi for each patient population, and emerging preclinical and clinical evidence in the field. Given the limited availability of randomized clinical trials in these populations, observational cohort studies, pilot trials, mechanistic studies, meta-analyses, and ongoing clinical trials were considered for inclusion.

## 3 Underrepresented populations in SGLTi landmark trials

### 3.1 Amyloid cardiomyopathy

Amyloid cardiomyopathy is a restrictive cardiomyopathy that typically presents with progressive diastolic dysfunction. This form of infiltrative cardiomyopathy develops due to extracellular deposition of misfolded amyloid fibrils, typically composed of immunoglobulin light chains in amyloid light-chain amyloidosis or transthyretin protein that can be either acquired (ATTRwt-CM) or hereditary (ATTRv-CM) (21–24). The resulting cardiac morphology features a reduced ventricular diameter and diastolic dysfunction secondary to symmetric septal and ventricular wall thickening (25). This restrictive ventricular structure leads to HFpEF, which can decline to reduced ejection fraction over the disease course (26, 27).

With the emergence of disease-modifying therapies, specifically in ATTR-CM improved outcomes have been reported (24, 28–30). However, these treatments are not widely accessible, and patients continue to experience significant morbidity and mortality (31–33). Thus, supportive therapy with conventional HF treatments remains common (23). Recent ATTR-CM guidelines emphasize the poor tolerability of the conventional HF therapies, such as *β*-blockers, angiotensin-converting enzyme inhibitors, angiotensin II receptor blockers, and angiotensin receptor-neprilysin inhibitors, in this population, which relies on heart rate to preserve cardiac output (11, 34, 35). Therefore, loop diuretics and mineralocorticoid receptor antagonists remain the mainstay of HF therapy in ATTR-CM (23). However, their use is often limited by the risk of hypotension in these patients with autonomic dysfunction and restrictive physiology (11, 34, 35).

Unlike traditional therapies, SGLTi may not interfere with hemodynamics in ATTR-CM. These agents have not been associated with reductions in heart rate or stroke volume and rarely cause clinically significant hypotension (36, 37). They preferentially reduce extracellular volume improving fluid balance (38). More specifically, they promote decongestion of the interstitial compartment, in contrast to intravascular fluid decongestion observed with loop diuretic use (35, 39, 40). This mechanism can be particularly beneficial in ATTR-CM where patients often poorly tolerate intravascular volume variations, as the volume influences cardiac preload and ultimately the cardiac output in the setting of a relatively fixed stroke volume (35).

ATTR-CM often coexists with renal dysfunction and albuminuria (41, 42). Several mechanisms have been proposed to explain how SGLTi reduce albuminuria and hyperfiltration. One hypothesis suggests that SGLTi lower intraglomerular pressure by promoting afferent arteriolar vasoconstriction via natriuresis and increased sodium delivery to the macula densa (43). Other proposed pathways include a reset in the volume setpoint and reduction of sympathetic activation, occurring subsequent to the initial contraction of plasma volume caused by natriuresis, and reduced Na^+^/H^+^ exchanger activity (44). Furthermore, a “thrifty substrate” hypothesis proposed by Ferrannini et al. has been widely cited as a potential cardiorenal protective mechanism of SGLTi (45). This model suggests that SGLTi promote a metabolic shift from glucose to ketone body utilization in the heart, kidneys, and other organs. These ketone bodies, including *β*-hydroxybutyrate, serve as an optimal energy source and act as metabolic signals that modulate stress pathways, thereby reducing oxidative stress and inflammation in the kidneys (45, 46). This nutrient-deprived state has been linked to increased autophagy and mitochondrial health, leading to a decrease in reactive oxygen species (ROS) and inflammation (44).

SGLTi may also confer anti-arrhythmogenic benefits in patients with ATTR-CM, who are predisposed to arrhythmias secondary to underlying elevated cardiac filling pressures and amyloid infiltration of the cardiac conduction system (47, 48). In this population, conventional rate-control therapies such as *β*-blockers are often poorly tolerated, as heart rate is a major determinant of cardiac output in restrictive physiology, and a lower heart rate ultimately results in a reduced cardiac output (26, 48, 49). Clinical observations from other high-risk populations provide indirect support for a potential anti-arrhythmic effect of SGLTi. Among patients with diabetes hospitalized for myocardial infarction, SGLTi use was associated with a lower incidence of ventricular arrhythmias compared with controls (50). Similarly, a recent network meta-analysis showed a decrease in atrial fibrillation risk with the use of dapagliflozin among patients with diabetes and underlying comorbidities (51). Although the exact anti-arrhythmogenic mechanisms of the SGLTi remain incompletely defined, several pathways have been proposed. SGLTi may stabilize cardiomyocyte membranes by reducing calcium overload and improving calcium-sodium handling by inhibiting the Na^+^/H^+^ cardiac exchanger (52). In addition, reductions in preload and afterload may attenuate myocardial stretch and limit adverse remodeling (53). Moreover, SGLTi can have anti-inflammatory effects, possibly by inhibition of the NLRP3 inflammasome (54). Lastly, attenuation of sympathetic activation, optimization of mitochondrial function, promotion of autophagy, and suppression of profibrotic pathways have also been postulated to minimize the arrhythmogenic fibrotic substrate (55).

Observational studies support the role of SGLTi in patients with ATTR-CM and AL cardiac amyloidosis, demonstrating a generally favorable safety and tolerability profile, with urinary tract infections being the most commonly reported adverse event (36, 56, 57). SGLTi use has been associated with weight loss, improved volume status, lower diuretic dependence, reduced uricemia, and decreased N-terminal pro b-type natriuretic peptide (NT-proBNP) levels (36, 56–58). Notably, diuretic dose, NT-proBNP levels, and uricemia are predictors of mortality in patients with ATTR-CM (59–61). Thus, SGLTi may play a role in mitigating diuretic resistance by lowering uric acid levels and myocardial wall stress, as evidenced by reductions in NT-proBNP levels.

Additionally, SGLTi are associated with a decrease in risk of ischemic stroke and renal function deterioration in ATTR-CM patients (62, 63). In a meta-analysis of five studies and 9,766 participants, SGLTi use was associated with a 46% decrease in all-cause mortality, 61% decrease in CV mortality, 29% decrease in major adverse CV events, 37% decrease in HF hospitalizations, and 27% lower odds of cardiac arrhythmias (64). However, this current evidence is mainly based on small, single-center retrospective studies with limited follow-up times and should therefore be evaluated cautiously.

Reflecting this growing body of evidence, SGLTi have been incorporated into the 2025 JACC expert consensus guidance for the management of transthyretin amyloid cardiomyopathy (23). Currently, two prospective trials are investigating dapagliflozin in patients with ATTR-CM, primarily focusing on quality of life, 6-minute walk distance, and changes in NT-proBNP (NCT05795400, NCT07240844) (65, 66). An overview of ongoing trials evaluating SGLTi in underrepresented populations is provided in Table 1.

**Table 1:** Outline of ongoing clinical trials of SGLTi in individuals with transthyretin amyloid cardiomyopathy, hypertrophic cardiomyopathy, adult congenital heart disease, heart transplant, and LVAD.

Population	Clinical trial identifier	Country/estimated size	Study design/study drug	Trial phase/status	Primary outcome(s)	Key secondary outcome(s)
Transthyretin Amyloid Cardiomyopathy	NCT05795400 (65)	Russia (*n* = 50)	Interventional/Dapagliflozin	Phase not applicable/Active not recruiting	Change in KCCQ-CSS; effect on 6MWD; effect on NT-proBNP	Cardiovascular death+number of hospitalizations due to heart failure; change in echocardiographic parameters; change in speckle-tracking echocardiographic parameters
	NCT07240844 (66)	South Korea (*n* = 68)	Interventional/Enavogliflozin	Phase 4/Not yet recruiting	Change in KCCQ-CSS	6MWD; NT-proBNP; body weight; body fat percentage; extracellular water ratio; incidence of worsening heart failure events or death
Hypertrophic Cardiomyopathy	NCT06433050 (95)	United States (*n* = 26)	Interventional/Sotagliflozin	Early phase 1/Recruiting	Treatment-related adverse events; intracavitary left ventricular pressure gradient; new occurrence of cardiac arrhythmia; peak oxygen consumption; stroke volume augmentation	Left ventricular ejection fraction; global longitudinal strain; E/E’ ratio; maximal left ventricular wall thickness; KCCQ-CSS; NT-proBNP; serum metabolites; daily step counts
	NCT05182658 (96)	Poland (*n* = 250)	Interventional/Empagliflozin	Phase 3/Unknown status	Change in peak VO₂	–
	NCT06481891 (97)	International (*n* = 500)	Interventional/Sotagliflozin	Phase 3/Recruiting	Change in KCCQ-CSS	≥1 NYHA class improvement; KCCQ Total Symptom Score
	NCT06580717 (193)	South Korea (*n* = 200)	Interventional/Empagliflozin	Phase 4/Recruiting	Change in LV diastolic reserve (*Δ*e′, stress Echocardiogram)	CPET parameters; NT-proBNP; troponin-T; KCCQ-CSS; NYHA; arrhythmic burden on 24-hour ambulatory electrocardiogram
	NCT06401343 (194)	China (*n* = 94)	Interventional/Empagliflozin	Phase 4/ Recruiting	Peak VO₂ (CPET)	KCCQ-CSS; HCMSQ-SoB; NYHA; NT-proBNP; diastolic echocardiographic parameters
	NCT07294495 (195)	China (*n* = 150)	Interventional/Henagliflozin	Phase unapplicable/Not yet recruiting	Change in active fibroblast activity (FAPI PET/CMR)	Change in myocardial FAPI maximum standardized uptake value; change in FAPI activity volume percentage; change in LVEF; change in left ventricular mass index; change in late gadolinium enhancement extent, change in global longitudinal strain; change in 6MWD; change in NT-proBNP; change in quality of life total score
Adult Congenital Heart Disease (ACHD)	NCT06932081 (134)	International (*n* = 400)	Observational	Phase unapplicable/Recruiting	Prescription patterns of SGLT2i	Side effects; SGLT2i discontinuation; mortality; hospitalizations; weight; blood pressure; heart rate, saturation; electrolytes; creatinine; eGFR; glucose; HbA1c; transthoracic echocardiography parameters; exercise parameters
	NCT06260059 (126)	United States (*n* = 40)	Interventional/Empagliflozin	Phase 4/Recruiting	Change in ventricular ejection fraction; change in T1 mapping; change in global strain; change in global longitudinal strain; change in functional exercise capacity; number of participants hospitalized for cardiac reasons or heart transplantation; all-cause mortality	Change in inflammatory serum markers; change in functional neuropsychological testing; change in NYHA class; change in patient-reported outcomes measurement information system; change in KCCQ; change in neuro-quality of life
	NCT05580510 (127)	Mexico (*n* = 160)	Interventional/Empagliflozin	Phase 3/Unknown status	Change in systemic ventricular EDVi/ ESVi	KCCQ; pulmonary congestion; 6MWD; LVEF; NT-proBNP; systemic venous congestion by VExUS grading system
	NCT05897489 (128)	France (*n* = 100)	Observational	Phase unapplicable/Recruiting	NYHA class/ NT-proBNP	SGLT2i prescriptions; plasma creatinine; hyperkalemia; adverse events; overall heart failure survival
Repaired Tetralogy of Fallot	NCT06668389 (130)	China (*n* = 106)	Interventional/Dapagliflozin	Phase 4/Recruiting	Peak VO₂ (CPET)	Echocardiographic parameters including right ventricular dimension/function and pulmonary/tricuspid regurgitation; cardiopulmonary test parameters; cardiac biomarkers; Minnesota Living with Heart Failure Questionnaire/KCCQ; time of occurrence of clinical events; occurrence of adverse events
Fontan Circulation	NCT06762964 (132)	United States (*n* = 27)	Interventional/Dapagliflozin	Phase 2/Recruiting	Pulmonary capillary wedge pressure	Right atrial pressure; pulmonary artery pressure; cardiac output; peak oxygen consumption during exercise; change in plasma volume; change in blood volume; SF-36 quality of life questionnaire; change in fat mass; change in fat free mass
	NCT06955260 (131)	International (*n* = 410)	Interventional/Empagliflozin	Phase 3/Not yet recruiting	Hierarchical endpoint of death, transplant, or MCS	All-cause death; listing for heart transplantation or initiation of MCS; Sustained VT/aborted SCD; HF hospitalization; ≥5-point KCCQ-CSS change; 6MWD
	NCT05741658 (133)	United States (*n* = 29)	Interventional/Dapagliflozin	Phase 4/Enrolling by invitation	Peripheral venous pressure	Total body water; CPET measured peak VO₂/ oxygen pulse/ ventilator efficiency slope/ oxygen uptake efficiency slope; peripheral venous pressure at exercise; ACHD PRO metric score
Heart Transplant	NCT06625073 (196)	United States (*n* = 200)	Interventional/Empagliflozin	Phase 4/Recruiting	Urinary albumin-to-creatinine ratio; incidence of treatment-emergent adverse events	eGFR; HbA1c; fasting blood glucose; body weight; systolic and diastolic blood pressure; hsCRP; NT-proBNP; hemoglobin; hematocrit; serum transferrin saturation; 6MWD; KCCQ-12; serum tumor necrosis factor-alpha; serum interleukin-6; serum interleukin-1β
	NCT05321706 (157)	International (*n* = 430)	Interventional/Dapagliflozin	Phase 3/Recruiting	eGFR slope	Body weight; HbA1c; proteinuria
	ACTRN12622000978763 (197)	Australia (*n* = 100)	Interventional/Empagliflozin	Phase 2-3/Recruiting	Change in HbA1c or fructosamine	LV fibrosis and mass (CMR); eGFR; hospitalizations; all-cause mortality
	NCT06147271 (198)	Brazil (*n* = 80)	Interventional/Empagliflozin or Dapagliflozin	Phase 2/Enrolling by invitation	Cardiac allograft vasculopathy	CV and all-cause mortality; CV hospitalization; worsening eGFR
Left Ventricular Assist Device (LVAD)	NCT05278962 (186)	United States (*n* = 32)	Interventional/Empagliflozin	Phase 4/Completed	Ramp stages to achieve hemodynamic optimization	–

6MWD, 6-minute walk distance; ACHD, adult congenital heart disease; CMR, cardiac magnetic resonance; CPET, cardiopulmonary exercise testing; eGFR, estimated glomerular filtration rate; FAPI PET/CMR, fibroblast activation protein inhibitor positron emission tomography/cardiac magnetic resonance; HbA1c, glycated hemoglobin; HCMSQ-SoB, hypertrophic cardiomyopathy symptom questionnaire–shortness of breath; hsCRP, high-sensitivity C-reactive protein; KCCQ, Kansas City Cardiomyopathy Questionnaire; KCCQ-12, 12-item Kansas City Cardiomyopathy Questionnaire; KCCQ-CSS, Kansas City Cardiomyopathy Questionnaire Clinical Summary Score; LV, left ventricle; LVEF, left ventricular ejection fraction; LVAD, left ventricular assist device; MCS, mechanical circulatory support; NT-proBNP, N-terminal pro-B-type natriuretic peptide; NYHA, New York Heart Association; peak VO₂, peak oxygen consumption; PRO, patient-reported outcome; SCD, sudden cardiac death; SGLTi, sodium–glucose cotransporter inhibitors; VExUS, venous excess ultrasound; VT, ventricular tachycardia.

### 3.2 Hypertrophic cardiomyopathy

HCM is the most common genetic cardiomyopathy, with an estimated prevalence of 1 in 500 in the general population (67). It is characterized by left ventricular hypertrophy that is not secondary to other co-existing medical conditions (68). Hypertrophic obstructive cardiomyopathy (HOCM) is a subtype of HCM, accounting for approximately 60% of cases, and is characterized by dynamic left ventricular outflow tract obstruction resulting from septal hypertrophy and systolic anterior motion of the mitral valve, which can exacerbate symptoms and increase the risk of secondary mitral regurgitation (69).

Pathogenic genetic variants are identified in approximately 30% of cases (68). The mutations that drive HCM include alterations in genes encoding sarcomeric proteins, the beta-myosin heavy chain, and myosin binding protein C3 (70, 71). Although the resulting pathophysiology is complex, mutated proteins generally have altered calcium affinity in myofilaments and structural abnormalities that lead to hypercontractility and impaired diastolic relaxation. Over time, these features contribute to myocyte hypertrophy, interstitial fibrosis, and coronary microvascular dysfunction, which in turn worsen diastolic dysfunction, while left ventricular ejection fraction is preserved or supranormal (70). In fact, nearly half of HCM patients meet the criteria for a HFpEF diagnosis (72). Furthermore, patients with HCM are also at increased risk of arrhythmias due to conductive heterogeneity secondary to fibrosis and myocyte disarray, altered protein expression, increased Na^+^ and L-type Ca^2+^ currents, decreased K^+^ currents, and abnormal adrenergic signaling (73–75). Atrial fibrillation and ventricular tachycardia are the most commonly observed arrhythmias in patients with HCM (76, 77).

SGLTi are potential candidates for management of HCM because of their potential to improve diastolic function, reduce hypercontractility, hypertrophy, and interstitial fibrosis. Preclinical evidence suggests that SGLTi may aid cardiac relaxation through several mechanisms. Firstly, SGLTi have been found to modulate the Na+/H + exchanger (NHE-1) and the sodium channel Na_v_1.5 in rabbit and mouse cardiomyocytes, thereby reducing cytosolic Ca^2+^ and Na^+^ levels, which increases contractility (52, 78). Similarly, in rat models of T2DM, SGLTi were found to reduce L-type Ca^2+^ conduction and cardiomyocyte shortening, consistent with reduced hypercontractility (79). In preclinical models, empagliflozin increased phosphorylation of cardiac troponin I, cardiac myosin-binding protein C, and phospholamban, thereby improving myocardial relaxation *in vivo* (80–82). Additionally, in a tissue-engineered *in vitro* model of HCM, SGLTi rapidly improved myocardial relaxation by modulating the Na^+^/Ca^2+^ exchange current (83).

Beyond effects on hypercontractility, preclinical evidence suggests that SGLTi may reduce myocardial fibrosis and associated diastolic dysfunction also through anti-inflammatory mechanisms (84). Consistently, human cardiac fibroblasts exhibited reduced proliferation and migration when treated with SGLTi (85), and monocytes exposed to SGLTi showed increased polarization to an anti-inflammatory M2 phenotype (86). The mechanism by which SGLTi reduce fibrosis is linked to the modulation of the AMPK and TGF-*β* inflammatory signalling pathways (87). Likewise, in rodent cardiac fibroblasts, dapagliflozin attenuated fibroblast activation and reduced collagen production in response to high glucose levels, also via an AMPK-dependent pathway (88). With regard to the TGF-*β* pathway, treatment of human cardiac fibroblasts with empagliflozin promoted a quiescent phenotype resistant to TGF-*β* signalling (89), and in a rodent model, empagliflozin reduced scar formation, cardiac fibrosis, and TGF-*β* expression after MI (90).

Leading the translation of these promising preclinical results to the clinic, a few recent studies have demonstrated potential benefits of SGLTi in HCM, notably in patients with diabetes and concurrent HCM. In a large observational cohort, use of SGLTi was associated with reduced all-cause mortality, HF hospitalizations, risk of sudden cardiac death, and incidence of ischemic stroke in diabetic adults with HCM (91). Similarly, in patients with diabetes and non-obstructive HCM, SGLTi were associated with improved diastolic function and a favorable safety profile (92), and real-world data indicate that SGLT inhibitor use in HCM is associated with fewer CV symptoms and lower mortality (93). A *post hoc* analysis of the SCORED trial showed that sotagliflozin was associated with a significant reduction in CV mortality, HF events, and a significant reduction in the composite of major adverse CV events plus HF hospitalizations in patients with left ventricular hypertrophy without hypertension (94).

Of note, the aforementioned studies are observational in design (87–89), with all but one (88) conducted retrospectively. Furthermore, two of the studies (87, 88) focus specifically on SGLT2i use in patients with both diabetes mellitus and HCM. Accordingly, these initial findings have significant limitations, including potential residual confounding despite propensity score matching, selection bias, and limited generalizability to patients without other indications for SGLT2i (e.g., diabetes mellitus), and relatively short follow-up that may be insufficient to capture long-term HCM trajectories. Therefore, these observations remain hypothesis-generating and require confirmation in randomized clinical trials.

To date, there are no published randomized clinical trials on SGLTi in HCM, but several randomized, placebo-controlled studies are currently underway to assess their efficacy. These include SONATA-HCM (NCT06481891), a multicenter trial evaluating the effects of sotagliflozin in both obstructive and non-obstructive HCM. Additional studies include SOTA-CROSS (NCT06433050), a crossover trial of sotagliflozin in non-obstructive HCM, and EMPA-REPAIR (NCT05182658), which is investigating empagliflozin in patients with HCM (Table 1) (95–97).

### 3.3 Congenital heart disease

CHD represents the most prevalent congenital malformation in newborns, encompassing an array of structural abnormalities, including left-to-right shunts, conotruncal defects, single ventricle circulation, and valvular disorders (98–100). Multiple gene mutations have been implicated in the disease etiology, including variants affecting MYH6, MYH7, ACTC1, NR2F2, and NOTCH1, along with contributions from environmental factors (100–102). As these individuals grow older, they are subject to a higher risk of HF, which is the leading cause of mortality in the adult CHD (ACHD) population (103, 104). The pathophysiology of HF in this population is driven by hemodynamic abnormalities related to altered cardiac anatomy, which ultimately can lead to myocardial ischemia, scarring, and hypertrophy (100, 105).

Surgery remains the cornerstone of CHD management. Despite surgical correction, many patients remain at risk of developing HF, even though the risk is lower than that of individuals with uncorrected defects (106–108). For instance, after the atrial switch procedure, the morphologic right ventricle supports the systemic circulation and is exposed to chronic pressure overload, predisposing it to progressive dysfunction over time (109, 110). Medical therapy for HF in ACHD is challenging and must be individualized in this heterogeneous population, which is often preload-sensitive and at increased risk of bradyarrhythmias and other rhythm disturbances. GDMT for HF is commonly used (103, 105, 107, 111), although the evidence is mostly limited to retrospective studies (112).

SGLTi use may be relevant in the CHD population, given their association with favorable hemodynamic effects and potential attenuation of pressure-related myocardial fibrosis. SGLTi mildly reduce afterload, which is the primary mechanism of HF in the setting of systemic right ventricle, while the effect on preload is most pronounced acutely but returns to baseline within 12 weeks as compensatory mechanisms establish a new steady state (113). In rodents, empagliflozin has been linked to reductions in right ventricular hypertrophy and fibrosis, with improvements in pulmonary hypertension (114). Hence, SGLTi may have broad applicability across distinct CHD phenotypes, including systemic right ventricular failure due to chronic pressure overload and Fontan circulation, which is highly sensitive to changes in pulmonary vascular resistance.

In a retrospective study of 174 individuals with various ACHD and concomitant HF, a treatment with SGLTi was associated with a threefold reduction in HF hospitalizations over a six-month follow-up period (RR 0.30, 95% CI 0.14–0.62) (115). In the same study, 10.3% reported side effects, of which 6.9% led to discontinuation of SGLTi (115). In a meta-analysis of eight studies and 289 participants with ACHD and concomitant HF, the initiation of SGLTi led to improvements in New York Heart Association (NYHA) functional class, NT-proBNP levels, and systolic blood pressure (116). In patients with single ventricular failure or failing Fontan circulation, SGLTi has demonstrated a consistent favorable safety profile (117–121). As for the arrhythmia risk, which is partly increased due to underlying fibrotic foci, the addition of SGLTi to background sacubitril/valsartan therapy in patients with failing systemic right ventricle was associated with significant reductions in atrial and ventricular arrhythmias (122).

Imaging studies support these findings. In a single-arm study of 32 patients with single ventricle dysfunction, initiation of dapagliflozin was associated with improvements in fractional area change, global longitudinal strain, and the 6-Minute Walk Distance, as well as quality of life over six months of follow-up (123). Similarly, dapagliflozin improved fractional area change and free-wall global longitudinal strain in patients with a failing systemic right ventricle (124). Another small study of patients with single-ventricular failure showed a decrease in end-systolic area and free wall strain with SGLTi use, although no improvement in global longitudinal strain was seen (125).

Several limitations characterize the available retrospective studies in ACHD, as in the previous subgroups. The studies were prone to selection bias and the follow-up times varied widely in the individual studies and were often reported as limited. In the echocardiographic studies, ventricular function was assessed using heterogeneous parameters that were not consistently defined across studies. Moreover, ACHD represents a highly heterogeneous population, and the distribution of underlying congenital lesions differed substantially across studies.

Multiple ongoing clinical trials are expected to further define the role of SGLTi in this population (Table 1). Some studies are focusing on imaging endpoints (NCT06260059, NCT05580510) (126, 127), while others are evaluating functional endpoints, including peak oxygen uptake, quality of life, and 6-minute walk distance (NCT05897489, NCT06668389, NCT06955260) (128–131) or hemodynamic endpoints, including changes in pulmonary capillary wedge pressure and peripheral venous pressure (NCT06762964, NCT05741658) (132, 133). In addition, an ongoing observational study is assessing SGLTi prescribing patterns in CHD (NCT06932081) (134).

### 3.4 Heart transplant

Patients with advanced HF remain symptomatic despite optimal GDMT, impairing their daily functioning and leading to recurrent hospitalizations (11). Heart transplantation remains a definitive therapeutic option for selected patients in this subgroup, offering over a decade of overall survival and improved quality of life (135, 136). However, its use is limited by the scarcity of donor organs and post-transplantation complications (137). Early complications such as acute allograft rejection and primary graft dysfunction, alongside long-term sequelae including cardiac allograft vasculopathy (CAV), post-transplant diabetes mellitus (PTDM), hypertension, and malignancy, are major drivers of post-transplant morbidity (138).

SGLTi use may be advantageous after HTx by targeting key mechanistic pathways of CAV and PTDM. CAV pathophysiology involves both immune and non-immune processes, leading to pan-arterial intimal thickening of the epicardial and intramyocardial vessels (138). The concentric narrowing of the vasculature ultimately increases the risk of major adverse events in these individuals, including myocardial infarction and sudden cardiac death (139, 140). Preventive treatment with statins and early-stage treatment with proliferation signal inhibitors are implemented, but they do not fully halt progression, and once advanced vascular lesions develop, re-transplantation often remains the only therapeutic option (138), highlighting the need for additional preventive strategies (141).

In this context, there has been a renewed interest in calcium channel blockers and angiotensin-converting enzyme inhibitors for their role in suppressing arterial occlusion in CAV (141, 142). SGLTi warrant equal consideration, as they could potentially minimize intimal proliferation of vessels, given that they are proven to promote endothelial function (143). Preclinical studies suggest that SGLTi-mediated endothelial benefits arise from improvements in vasodilation, nitric oxide production, mitochondrial homeostasis, endothelial cell viability, angiogenesis, alongside reductions in pathological inflammation and oxidative stress (143). Emerging clinical data support these findings, as the use of SGLTi are associated with improvements in flow-mediated vasodilatation and pulse wave velocity (144). Furthermore, SGLTi have benefits in the management of risk factors associated with CAV, such as hypertension and hyperglycemia (138, 145, 146).

Nearly a fifth of HTx recipients develop PTDM, primarily attributable to pre-existing comorbidities, traditional diabetes risk factors, and the adverse effects of immunotherapy (147–149). The current PTDM management guidelines do not endorse one glucose-lowering treatment over another, given the lack of clinical data on the subject (150). In rodent models, an increase in SGLT-1 expression was seen in the jejunum following 14 days of tacrolimus intake and in SGLT-2 expression in the proximal renal tubule following three weeks of tacrolimus use (151, 152). The administration of empagliflozin for one week in these rodents resulted in a dose-dependent reduction of SGLT-2 expression in the proximal renal tubules, suggesting another pathway through which SGLTi may target PTDM (151, 153).

Additionally, SGLTi may help mitigate post-transplant anemia by stimulating erythropoietin (EPO) production and reducing inflammation (154). This can be important as nearly half of HTx recipients develop anemia following transplantation (155). The safety profile is also favorable, as SGLTi do not utilize CYP450 enzymes for their metabolism and therefore minimally interact with patients’ ongoing immunotherapy, including calcineurin inhibitors (156).

Two recent retrospective observational studies examined the use of SGLTi in HTx recipients. In a cohort of 21 HTx patients with T2DM, SGLTi were safe and well-tolerated, with no occurrence of diabetic ketoacidosis, genital mycotic infections, pancreatitis, or interactions with immunotherapy agents during a median follow-up of 9.1 months (146). During follow-up, patients reported significant weight loss, reduced insulin requirements, and a decrease in glycated hemoglobin (HbA1c), with approximately one-third no longer requiring insulin therapy by the end of the follow-up period.

Similarly, Cehic et al. studied the safety profile of SGLTi in 22 diabetic individuals with HTx and compared the results to those of 79 controls on metformin (147). SGLTi were associated with reductions in the required furosemide doses, body weight, and HbA1c compared to metformin after one year of follow-up. With regard to safety, adverse events were rare, renal function remained stable, and there were no genitourinary infections in the empagliflozin group.

These preliminary findings have limitations similar to those discussed in previous sections. These studies are observational and retrospective (146, 147). Furthermore, they focus on SGLT2i use in patients with both diabetes mellitus and HTx, leading to selection bias and limited generalizability. Finally, follow-up times (maximum 1 year) are insufficient for long-term HTx trajectories, including the development of CAV and PTDM.

The ongoing Phase 3 randomized trial, DAPAHRT (NCT05321706), is further investigating the use of dapagliflozin to stabilize kidney function in HTx recipients, who are at elevated risk of nephrotoxicity due to long-term calcineurin inhibitor exposure (Table 1) (157). The trial will also explore other outcomes of interest, such as cardiac health and new-onset diabetes.

### 3.5 Left ventricular assist devices

LVAD are widely used forms of mechanical circulatory support utilized in end-stage HF. LVADs can be employed as a bridge to transplantation therapy, a destination therapy, or, less commonly, as a bridge recovery (158, 159). The largest proportion of patients are directed to LVAD as destination therapy, as they are ineligible for a HTx, often due to end-stage comorbidities, advanced age, or psychological barriers (18, 159, 160). Bridge-to-transplant therapy is dedicated to those awaiting a HT, who may benefit from hemodynamic stabilization and optimization of their surgical candidacy (159).

LVAD improves overall survival and quality of life in patients with advanced HF by unloading the left ventricle, improving hemodynamics, and attenuating neurohormonal activation, ultimately leading to reverse ventricular remodeling (159, 161). Nonetheless, many patients continue to experience residual HF symptoms and remain at risk for recurrent hospitalizations and progression of cardiorenal disease. Moreover, LVAD is associated with complications, such as infections, stroke, bleeding, thrombosis, and right ventricular dysfunction. Consequently, management often requires careful optimization of volume status, pump speed calibration, and anticoagulation administration (162–165). Given these challenges, SGLTi pleiotropic effects may potentially benefit the LVAD population by improving hemodynamics and cardiorenal health and, indirectly, mitigating stroke risk.

Moreover, volume status shifts, such as overdiuresis and hemodynamic changes secondary to device implantation, can precipitate adverse events, including pump suction episodes and right ventricular dysfunction (166). SGLTi reduce plasma volume, blood pressure, preload, and afterload by osmotic diuresis and natriuresis, and may thus possibly complement loop diuretic therapy. Although data in LVAD patients are limited, SGLTi have been associated with a reduction in pulmonary artery pressure and improved right ventricular function (167–169). As such, SGLTi may be a useful adjunct to optimize volume status and hemodynamics in LVAD patients but requires further prospective evidence.

In addition, the nephroprotective effects of SGLTi may be particularly relevant for patients with LVAD, in whom renal dysfunction and progression of chronic kidney disease (CKD) are frequently observed (170). Consistent with this, multiple randomized clinical trials have shown that SGLTi slow CKD progression and acute kidney injury risk in patients with CKD, independent of diabetes status and glycemic control (153, 171–173). Finally, both ischemic and hemorrhagic stroke remain a significant risk in LVAD patients (174). In non-LVAD populations, SGLTi use has been associated with a 20% risk reduction of ischemic stroke in patients with diabetes and atrial fibrillation (175). Although the exact mechanisms remain under investigation, experimental evidence suggests that SGLTi may attenuate atherosclerotic progression by reducing oxidative stress and inflammation, as well as by suppressing foam cell formation and platelet activation (175, 176). Furthermore, endothelial dysfunction is a key contributor to both ischemic and hemorrhagic stroke, driving vasoconstriction, thrombosis, inflammatory cell infiltration, and disruption of vascular integrity (177, 178). SGLTi may in theory mitigate stroke risk by improving endothelial function through reductions in oxidative stress and inflammation (171, 179–181).

Several studies have investigated the safety profile of SGLTi in LVAD patients. Overall, these studies report a favorable safety profile and findings consistent with the potential benefits outlined above. A retrospective study of 1,312 patients with LVAD treated with and without SGLTi found that SGLTi users had significantly reduced odds of adverse outcomes, including all-cause mortality, all-cause hospitalization, and acute HF exacerbation, compared to propensity-matched non-users (182). Several smaller retrospective studies echo these findings (163, 183–185). Regarding renal outcomes, available retrospective analyses have not demonstrated a clear renal benefit associated with SGLTi therapy in patients with LVADs (163, 184, 185). The available studies are limited by small sample sizes, short follow-up durations, and retrospective design. Data regarding the type of LVAD implanted was often limited and there was variability in the timing of SGLTi initiation.

Results from the recently completed prospective randomized trial with SGLTi in LVAD patients will assess the number of ramp stages required to reach hemodynamic optimization (NCT05278962) (186). However, additional prospective, randomized, placebo-controlled studies are needed to fully characterize the efficacy of SGLTi in this population. Future trials should consider incorporating hemodynamic endpoints (e.g., LVAD ramping, volume status, and ventricular function), alongside renal outcomes (e.g., glomerular filtration rate, creatinine), and endothelial metrics (e.g., flow-mediated dilation, asymmetric dimethylarginine).

## 4 Safety considerations

A few safety considerations should be outlined when initiating SGLTi in these populations. Patients with amyloid cardiomyopathy, HOCM, Fontan physiology, or LVAD support may be particularly sensitive to changes in volume status. Although SGLTi produce only modest osmotic diuresis and natriuresis compared with loop diuretics (35, 39, 40), they may still contribute to volume depletion or symptomatic hypotension, particularly when combined with background diuretic therapy such as loop diuretics or mineralocorticoid receptor antagonists (187–189). Reassuringly, retrospective studies in amyloid cardiomyopathy have reported low rates of discontinuation or hypotension (36, 37), and large trials in HFrEF, have not demonstrated an increased risk of hypotension with SGLTi use (190). Nonetheless, careful monitoring of blood pressure, symptoms of dizziness or syncope, and overall volume status is therefore recommended after therapy initiation, and adjustment of concomitant diuretic therapy may occasionally be required.

Even a transient decline in estimated glomerular filtration rate during the first weeks of SGLTi therapy is common; initiation or use usually does not necessitate alteration of the frequency of kidney function monitoring (191). Although uncommon, euglycemic ketoacidosis has been reported with SGLTi therapy, particularly in the setting of acute illness, reduced oral intake, or during the perioperative period. Transplant recipients and patients supported with LVADs, are exposed to these triggers more frequently and may require temporary interruption of therapy during acute illness or procedural periods, with discontinuation commonly recommended 3–4 days before surgery (192). In addition, HTx recipients on immunosuppressive regimens have heightened susceptibility to infection, and SGLTi are associated with an increased risk of genital mycotic infections, and less commonly urinary tract infections. Reassuringly, early pilot studies suggest these agents may still be well tolerated with appropriate monitoring (147).

## 5 Conclusion

SGLTi are promising candidates for therapeutic and symptomatic treatment across a wide range of CV diseases and cardiomyopathies. While their efficacy in HFrEF and HFpEF is well-established, observational studies are beginning to elucidate their additional potential benefits in populations historically underrepresented in major clinical trials, including patients with amyloid cardiomyopathy, HCM, CHD, HTx, and LVAD. In particular, the anti-inflammatory, antifibrotic, diuretic, and cardiorenal-protective pleiotropic effects of SGLTi may be especially advantageous in patients who are highly sensitive to hemodynamic changes. Nevertheless, confirmation of these findings will require adequately powered, long-term randomized controlled trials. The results of ongoing and future trials will be critical in defining the role of SGLTi within these complex and heterogeneous patient populations.
